# An e-consent framework for tiered informed consent for human genomic research in the global south, implemented as a REDCap template

**DOI:** 10.1186/s12910-022-00860-2

**Published:** 2022-11-24

**Authors:** Tsaone Tamuhla, Nicki Tiffin, Taryn Allie

**Affiliations:** 1 Division of Computational Biology, Integrative Biomedical Sciences Department, Faculty of Health Sciences, University of Cape Town, Cape Town, South Africa; 2 Wellcome Centre for Infectious Disease Research in Africa, Institute of Infectious Diseases and Molecular Medicine, University of Cape Town, Cape Town, South Africa; 3 South African National Bioinformatics Institute, University of the Western Cape, Cape Town, South Africa

**Keywords:** Tiered informed consent, participant information, REDCap

## Abstract

Research involving human participants requires their consent, and it is common practice to capture consent information on paper and store those hard copies, presenting issues such as long-term storage requirements, inefficient retrieval of consent forms for reference or future use, and the potential for transcription errors when transcribing captured informed consent. There have been calls to move to electronic capture of the consent provided by research participants (e-consent) as a way of addressing these issues. A tiered framework for e-consent was designed using the freely available features in the inbuilt REDCap e-consent module. We implemented ‘branching logic’, ‘wet signature’ and ‘auto-archiver’ features to the main informed consent and withdrawal of consent documents. The branching logic feature streamlines the consent process by making follow-up information available depending on participant response, the ‘wet signature’ feature enables a timestamped electronic signature to be appended to the e-consent documents and the ‘auto-archiver’ allows for PDF copies of the e-consent documents to be stored in the database. When designing the content layout, we provided example participant information text which can be modified as required. Emphasis was placed on the flow of information to optimise participant understanding and this was achieved by merging the consent and participant information into one document where the consent questions were asked immediately after the corresponding participant information. In addition, we have provided example text for a generic human genomic research study, which can be easily edited and modified according to specific requirements. Building informed consent protocols and forms without prior experience can be daunting, so we have provided researchers with a REDCap template that can be directly incorporated into REDCap databases. It prompts researchers about the types of consent they can request for genomics studies and assists them with suggestions for the language they might use for participant information and consent questions. The use of this tiered e-consent module can ensure the accurate and efficient electronic capture and storage of the consents given by participants in a format that can be easily queried and can thus facilitate ethical and effective onward sharing of data and samples whilst upholding individual participant preferences.

**Supplementary Information:**

The online version contains supplementary material available at 10.1186/s12910-022-00860-2.

## Background

Research involving human subjects generally requires voluntary participation and signed consent from participants granting researchers permission to use their biological and/or health data [1–3]. To facilitate this process, researchers are required to provide detailed and transparent information about their research in a format that allows eligible participants to make informed decisions about whether to volunteer to participate in the research [3–6]. The informed consent process has frequently been criticised for not being participant-centred but rather more focused on meeting legal and regulatory requirements resulting in consent forms which use complex technical terms which lay persons cannot understand—especially those in vulnerable populations with limited health literacy [3–5]. There is therefore a need to improve the informed consent process by using consent documents that are straightforward and use easy-to-understand language to ensure that participants give truly informed consent [3, 4, 7].

It is common practice to capture consent information on paper and store those hard copies, and while this has its advantages, it presents issues such as long-term storage requirements and inefficient retrieval of consent forms for reference or future use [7–9]. In addition, for tiered informed consent where participants answer a variety of questions about allowed data or specimen use, paper-based consents are inefficient and impractical for determining whose data or which specific data elements have consent for onward sharing, meta-analyses or sharing in aggregated form [10]. Participants may also express a variety of preferences for future contact and/or feedback of findings from the research programme. While it is possible to transcribe this information from hard copies into electronic format, this is time-consuming and prone to data capture error, which might lead to unacceptable transgression of participants’ choices about how their data and specimens might be used [5]. There have been calls to move to electronic capture of the consent process (e-consent) as a way of addressing these issues [7, 8, 10, 11]. However, there has been slow uptake of e-consent because of technical, legislative, and regulatory barriers to setting up and implementing e-consent platforms. These include concerns about data security, legal validity of electronic signatures, and initial development costs [8, 9, 11, 12].

Researchers can find designing informed consent processes overwhelming and may not know how to implement them or what content is required. Using our experience in conducting tiered informed consent in South Africa, we have designed a REDCap-based electronic tiered informed consent framework that can aid in reducing barriers to uptake and implementation of e-consent in low- and middle-income countries. The framework is designed to improve the informed consent process for both participants and researchers involved in human genomic research, firstly by providing a comprehensive list of information for researchers to include in the consent documents, thus providing a tool which they can use as a check list to ensure that all essential information is available; and secondly providing researchers with ready-to-use, downloadable template consent documents which have been written in straightforward genomic research language that is easier for participants to understand. In this paper we present the content for the modules that can be used to construct the integrated participant information and consent form and describe how the REDCap template can be implemented to create study-specific tiered consent. A checklist that summarises the processes and consent modules is provided as Additional file 1.

The REDCap-based tiered e-consent module presented here facilitates the electronic capture of participants’ consent choices so no additional data entry is required and errors are kept to a minimum. We recommend that this process is undertaken by trained personnel who can accurately capture the preferences of the participants. We have also provided a REDCap database template so that researchers can easily incorporate this tiered informed consent module into their new REDCap research databases in a “ready-to-use” format, selecting elements and modifying the contents to fulfil their requirements without needing to develop new material de novo. For re-use of data and specimens, the captured information can be rapidly and easily queried to identify which resources have consent for other onward uses, and which participants might be re-contacted in the future for follow-up or related studies – thus facilitating efficient and ethical data-sharing and follow-up with participants where their consent has been given.

## Construction and content

### Setting up the tiered e-consent framework in REDCap

**Fig. 1:**
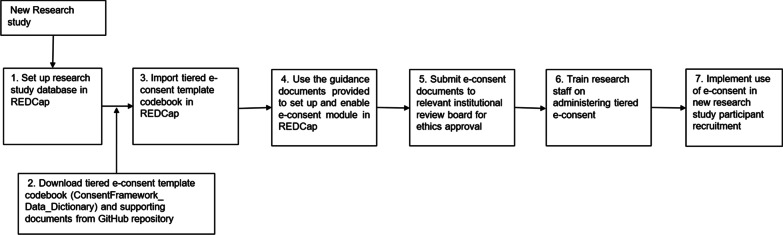
Flow diagram showing the workflow for setting up and implementing the tiered e-consent framework in REDCap for a new human genomic research study

Research Electronic Data Capture (REDCap) is a secure online databasing platform that allows production of generalisable data capture instruments for research [13]. REDCap has an inbuilt e-consent framework where consent is administered as a survey [14]. The tiered e-consent framework for genomic research was designed using tools in REDCap version 10.9.4 and is available as template data dictionary (ConsentFramework_Data_Dictionary) which researchers can download from GitHub (https://github.com/CIDRI-Africa/e-Consent-framework) and import into REDCap to set-up their own tiered e-consent module (Fig. 1). The tiered e-consent template is modular, allowing users to select elements which are suitable for their study. In addition, guidance documents which include a REDCap set-up guide, an instrument index which describes the data collection instruments available in the module and PDF copies of example data collection instruments are also available in the GitHub repository (see Additional file 2).

All consent documents used in a human genomics study need ethics approval before they can be used. The ethics review process for this REDCap based tiered e-consent module is like that of the paper-based consent because it does not contain any multi-media information such as videos. All the online tiered e-consent documents can be downloaded and submitted as PDFs to the institutional review board (IRB) and if required, a link can be set-up to give the IRB access to consent surveys online, on the REDCap platform. To successfully implement the tiered e-consent, research staff will need to be trained on how to navigate the REDCap platform and how to administer tiered informed e-consent.

### Tiered e-consent data collection instruments

**Fig. 2:**
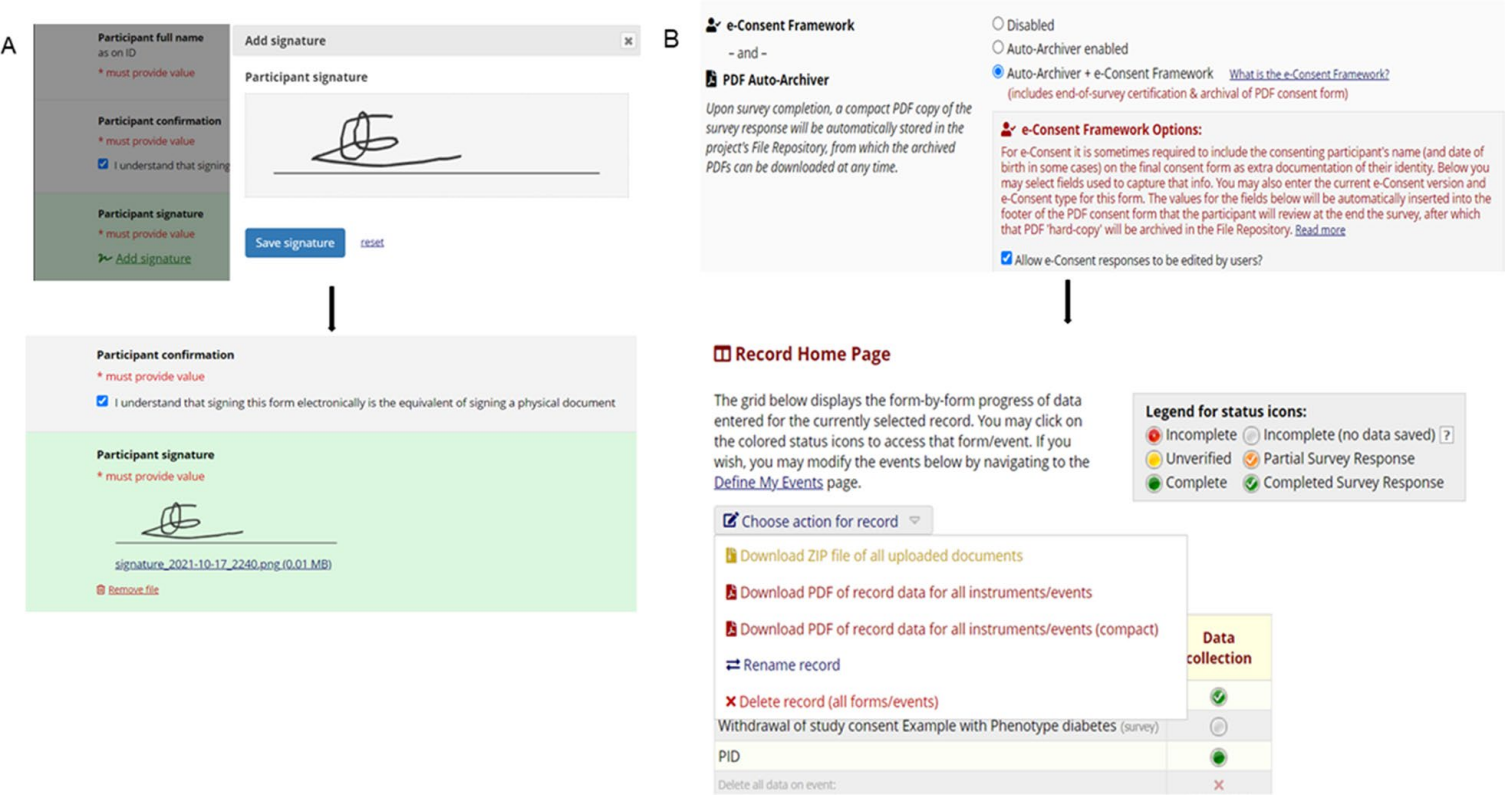
The **A** ‘wet signature’ and **B** ‘auto-archiver’ features in the tiered e-consent framework

The tiered e-consent module has three data capture instruments documents namely, the main consent and withdrawal of consent, which are both surveys, and an optional study meta data form. The inbuilt REDCap e-consent module has eight freely available features previously described by Lawrence et al. [14] which enhance the utility and security of the data capture instruments. For this tiered e-consent module we implemented ‘branching logic’, ‘wet signature’ and ‘PDF-consent document repository (auto-archiver)’ [14] to the main consent and withdrawal of consent documents. The branching logic feature streamlines the consent process by making follow-up information available depending on participant response and it was used in both the consent surveys. The ‘wet signature’ feature enables a timestamped electronic signature to be appended to the e-consent documents and the ‘auto-archiver’ allows for PDF copies of the e-consent documents to be stored in the database (Fig. 2) [14].

### Additional REDCap survey customisations

To ensure that tiered e-consent framework facilitates improved data quality, storage, retrieval and integrity, REDCap has additional customisations (see Additional file 3) which can be enabled for the consent surveys. When generating new records, the ‘*designate a secondary unique field*’ customisation allows the user to assign one of the variables such as the participant study ID as a unique value which cannot be duplicated. When this feature is enabled, each time that variable is entered it is checked in real time to ensure that it has not been assigned already. This will help with improving data quality as participants will not be assigned the same study ID especially in multi-site studies or where multiple people are carrying out consent simultaneously. When enabled, the ‘*display the Today/Now button for all date and time fields on forms/surveys*’ ensures that the current date or time will be set automatically by clicking a button. The ‘*set a custom record label’* feature allows another variable such as the participant study ID to be appended to the system generated numeric record name, to simplify the query and retrieval of individual participant records from the database. To ensure data integrity, three additional customisations namely ‘*require a reason when making changes to existing records*’, ‘*enable the data history pop up for all data collection documents’* and ‘*enable the field comment log or data resolution workflow (data queries)*’ can be enabled. These features described in detail in Additional file 3, ensure that any changes made to the consent documents after verification and signing are not only sanctioned but are recorded appropriately to ensure data integrity. In addition, user rights and permissions can also be set to determine who can add and/or edit records in the tiered e-consent framework.

### Language and layout of data collection instruments

**Fig. 3:**
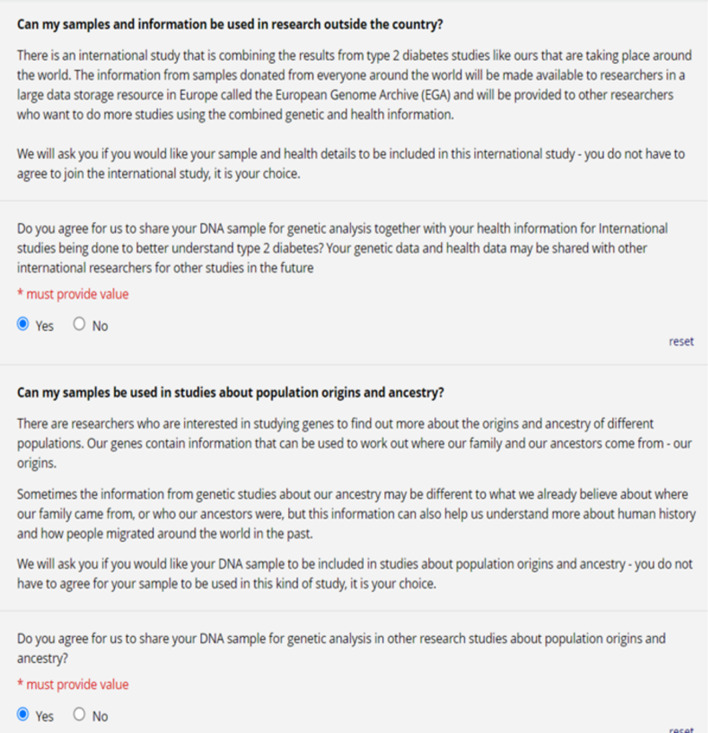
The layout of the consent was changed so that consent questions came directly after the corresponding participant information. This figure also shows the simple language used and how tiered informed consent was implemented in the e-consent framework

The content of the main consent was adapted from the tiered informed consent framework of Nembaware et al. [15] with some modifications, most notable of which was the addition of consent for the use of participant genomic data in population and ancestry studies which was excluded from that framework. Most consent documents have the same layout, where participant information is presented first, and consent questions are asked at the end or even on a separate document. When designing the content layout of the main consent document in the tiered e-consent framework, emphasis was placed on the flow of information to optimise participant understanding. This was achieved by merging the consent and participant information into one document where the consent questions were asked immediately after the corresponding participant information (Fig. 3). This format is intended to allow the participant to ask further questions and seek clarity on specific points before making a consent choice. In addition, we have provided example text for a generic human genomic research study on type 2 diabetes where the researchers are collecting both DNA and routine electronic health data from participants (Fig. 3). This text can be easily edited and modified to suit different research topics, and we recommend participant information and consent questions are modified and validated to suit each context in which this template is used.

## Utility of the platform

### E-consent platform

One of the barriers to the uptake and implementation of e-consent is the choice of a hosting platform and the costs associated with setting it up [9, 12]. This tiered e-consent platform was set-up in REDCap because REDCap already has an inbuilt e-consent framework which has been tested and shown to support various types of e-consent models [12, 14, 16, 17] and is freely available on a licence agreement to organisations that are part of the REDCap consortium [18]. The REDCap consortium currently comprises of more than 4000 institutions in 137 countries and membership has the added advantage of free access to technical support and improvements to the platform [14, 18]. In addition, using REDCap as the hosting platform, has the added advantage of having a single database for capturing and storing all research related data and this functionality was demonstrated for a Tuberculosis database (TBDBT) by Allie et al*.* [19].

### Administering tiered e-consent

One of the objectives of this tiered e-consent framework was to improve participant understanding of human genomics research so that they could make truly informed consent. So, while e-consent modules are commonly designed to be participant self-administered [12, 20–23], the main consent document in the tiered e-consent framework will be administered face-to-face by a trained member of the research team. This mode was preferred because it affords the participant the opportunity to ask questions if they seek clarity and numerous studies have shown that participants prefer to interact with the research team as this is associated with building rapport and establishing trust [4, 8, 9]. In addition, because this framework was developed for use in low and middle-income countries, a self-administered e-consent would not be practical. This is because REDCap is an online platform and surveys are sent to participants as a link. This would therefore potentially exclude participants who do not have access to a smart device or an internet connection and those who have limited digital literacy, particularly the elderly and those in rural areas [8, 11, 24].

### Data capture and storage

**Fig. 4:**
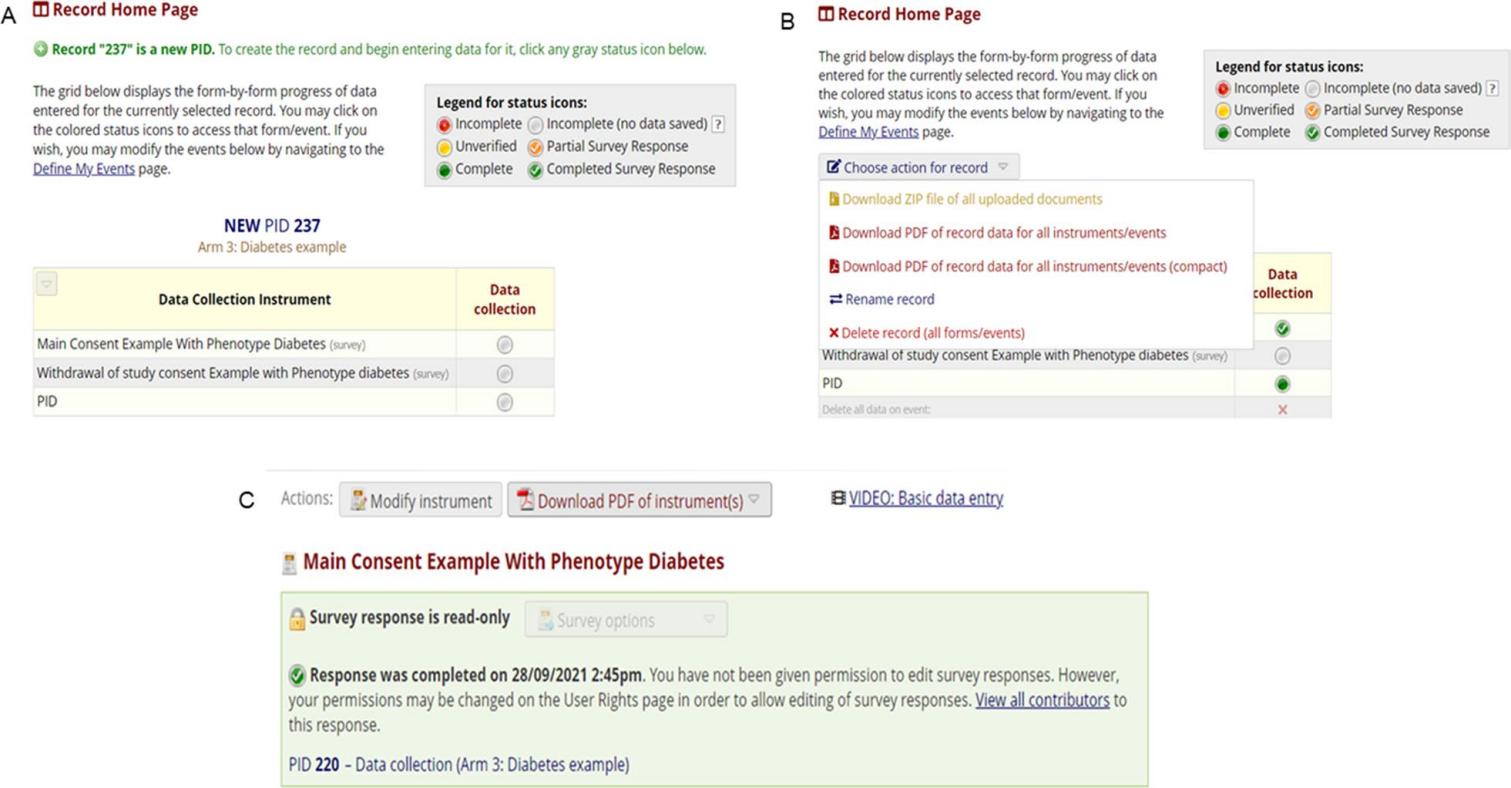
An overview of the records home page in REDCap. **A** Assignment of a PID to initiate collection of consent data, **B** dashboard showing the status of data collection in each instrument and **C** an example of user permission assignment

The main consent document uses tiered informed consent [15] and captures eleven different types of consent in one document (Table 1). The consents listed are the most common in human genomic research, but the list is not exhaustive, and users of this framework can choose which elements to include or leave out in their consent form based on their research needs. The ‘Add/Edit records’ function under the data collection page on the e-consent framework is used to initiate the consent process and launch the consent documents as surveys. REDCap automatically assigns a new consent survey with a unique record name (PID) which is numeric, system generated and cannot be changed (Fig. 4A). In addition, to ensure data quality and integrity, REDCap has mandated auto numbering for all survey instruments, so that users cannot manually name new records - a feature which ensures records do not share a PID.

**Table 1:** List of the type of consents that are available in the main consent of the tiered e-consent framework

Primary consent for collecting biospecimens and health data for specific disease in current study
Consent for access to medical records
Consent for return of individual results
Consent for return of individual results that are actionable and/or treatable
Consent for return of individual results that are NOT actionable and/or treatable
Consent for inclusion of individual data in genetic summary data
Consent for use of genetic and health data for future studies on specific disease
Consent for use of genetic and health data for future studies on other health conditions or related health processes
Consent to re-contact for future studies
Consent for use of genetic and health data in international studies
Consent for use of genetic data in population origins and ancestry studies

If the consent process is not completed in one sitting, the records page has a dashboard which shows the status of each record (Fig. 4B), and the current progress can be saved and concluded later. The PID is central to data retrieval, because once assigned it is linked to all data collection documents in the e-consent module for each participant. To retrieve an existing record, it is queried by PID and while this may be practical for a few records it will be impractical for projects with many participants. To mitigate this, a participant specific custom record label (see Additional file 4) such as the participant study ID can be appended to the PID allowing the user to retrieve individual participant records easily. The records home page also allows for records linked to a PID to be downloaded as PDFs and shared with those authorised to view them. To ensure data security and privacy, the consent documents in the tiered e-consent framework are strictly for collecting consent information and do not collect sensitive participant information such as demographic data or contact details. In addition, to ensure data integrity, REDCap has the functionality to assign user roles and permissions for accessing, editing and/or deleting existing records after they have been verified and signed by the participant (Fig. 4C).

### Data verification

**Fig. 5:**
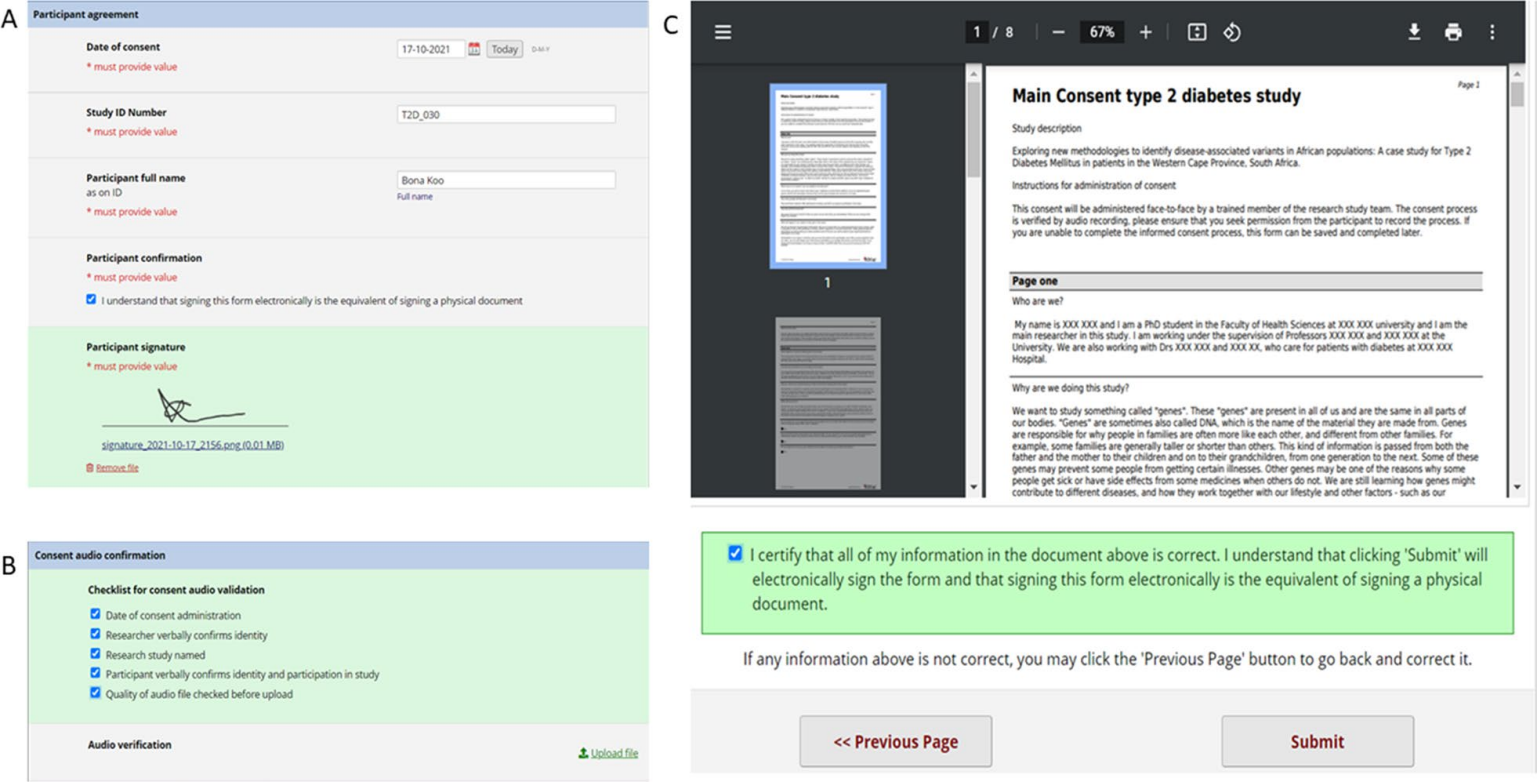
Verification and validation of the e-consent process by **A** electronic signature, **B** audio verification and **C** review and certification of the consent choices made by the participant before form submission

To meet legal and regulatory requirements all consent documents are validated by date-stamped electronic signature (Fig. 5A). In cases where an electronic signature is not legally recognised, the consent can still be done online, and the form downloaded, printed and signed by hand. The signed form or the signature itself can then be scanned and uploaded as an attachment alongside the signature field in the e-consent form. In this instance we would recommend archiving the signed paper forms in case they are required in the future. This will ensure that all consent data is still captured electronically directly into the REDCap database. For transparency, the e-consenting process will have two verification steps. The first is audio verification (Fig. 5B) for which participant permission will be sought before the consenting begins, and the audio file generated can also be uploaded and stored in REDCap. The second is through the ‘auto-archiver’ feature (Fig. 5C) which gives the participant an opportunity to verify that their choices were captured accurately before the consent is finalised.

### Data query and export

**Fig. 6:**
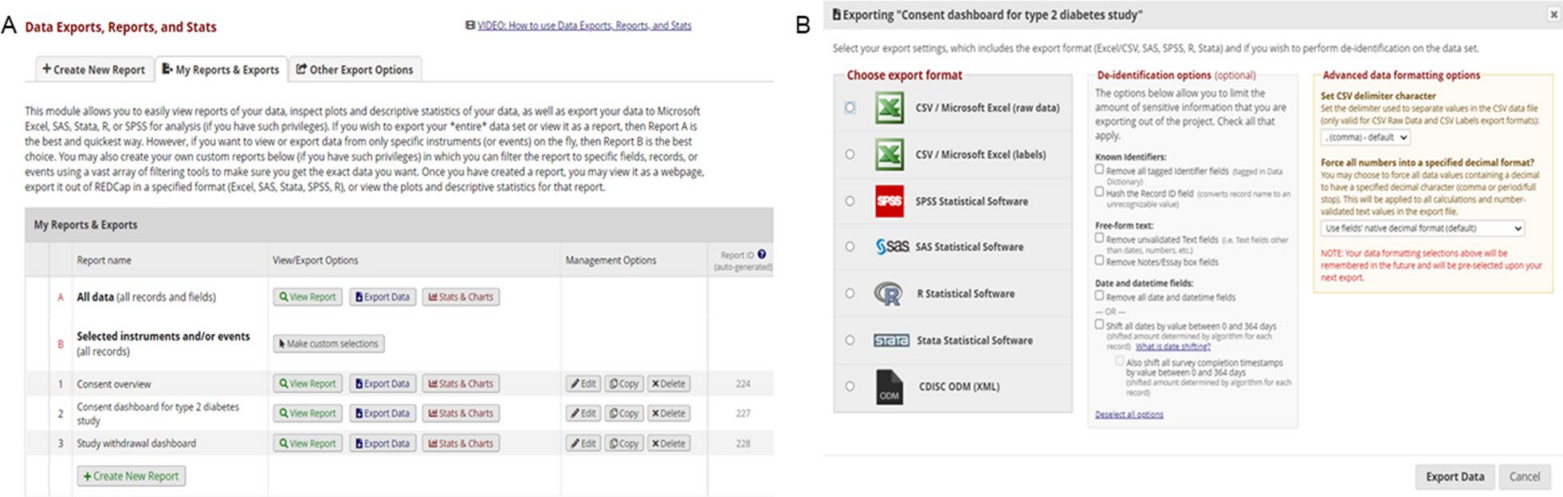
The data export, reports, and statistics page in REDCap. **A** An overview of all reports in the database and **B** the dashboard for the automated export of data from REDCap

All data that are entered into the tiered e-consent module are automatically stored in REDCap and can be viewed and downloaded from the Reports tab (Fig. 6A). REDCap automatically creates reports, but also allows for the customisation of reports to suit specific research needs by allowing users to select which data elements to include in each report. For the tiered e-consent module, we created two customised reports, being the consent dashboard (see Additional file 4) and the study withdrawal dashboard (see Additional file 5), which contain information on who has consented and/ or withdrawn from the study both at an individual level and for the entire study population. In addition, because this is tiered consent, the study population data is summarised for each type of consent covered in the main consent (see Additional file 6).

REDCap supports automated export of study reports, and the data can be downloaded in a format suitable to a selection of commonly used statistical packages (Fig. 6B). These reports also allow researchers to easily monitor the progress of their recruitment process in real time for in-house use and for submitting study progress reports to institutional ethics review boards. To protect participant privacy, there is an option to hide all tagged identifier fields and/or hash-to the record ID field. In addition, because the ‘data exports, reports and stats’ feature make it easy for researchers to query the database, identify consenting individuals and download their consent data this will facilitate ease of collaboration among researchers conducting human genomic studies.

### Withdrawal of consent

**Fig. 7:**
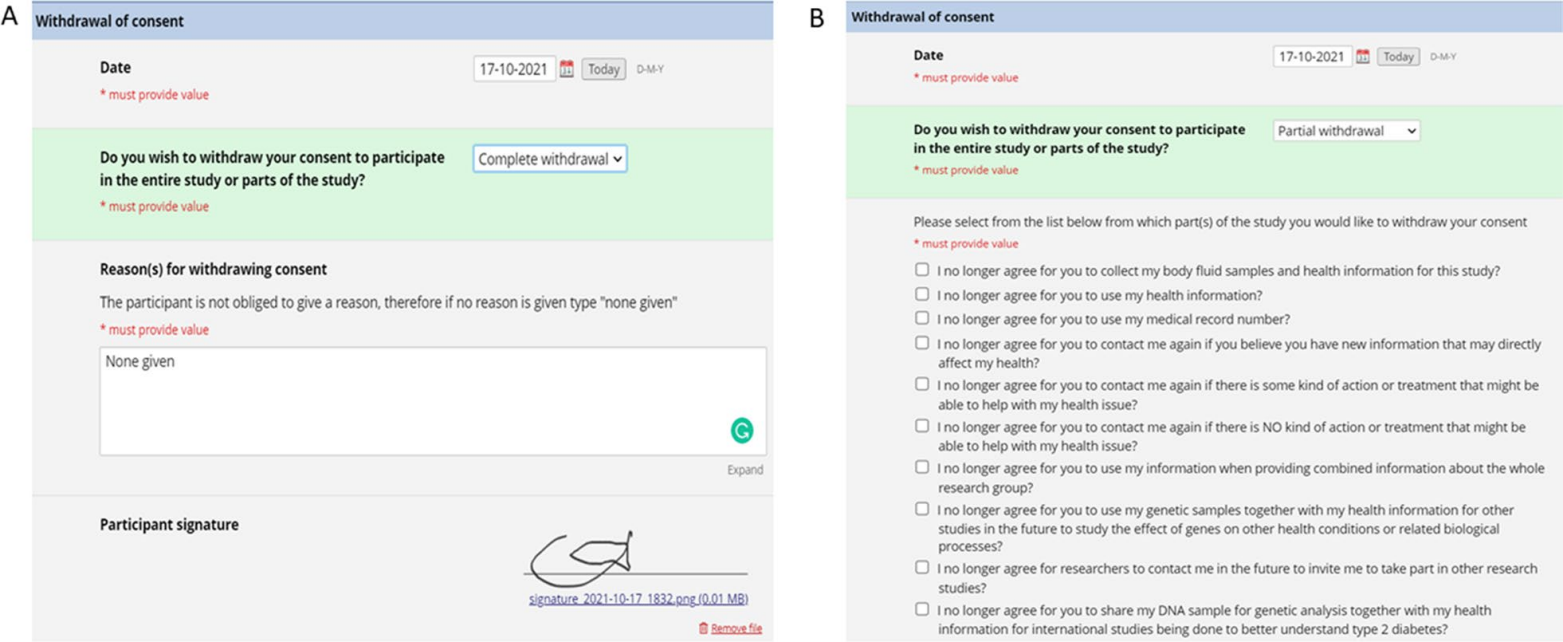
Implementation of ‘branching logic’ in the withdrawal of consent document. **A** Complete withdrawal and **B** partial withdrawal with the option for the participant to select for which elements they want to withdraw their consent

An important feature of voluntary participation in research is that participants can withdraw from the study whenever they wish. To accurately document participants who wish to withdraw their consent, the withdrawal of consent document is used. The consent can be partial or complete, and ‘branching logic’ (Fig. 7) is used to differentiate the two. If a participant selects to withdraw from the study completely (7A) then the next option is to provide a reason and then sign. However, if a participant wishes to withdraw only certain parts of their consent then a list of options opens, and the relevant ones are selected. Following this, the rest of the process is the same as for complete withdrawal.

## Discussion

Part of ethical research is ensuring that we make the best use of collected data and specimens, in line with the permissions that are given by participants. The use of broad consent has created some barriers to onward use of data, as it is not always clear exactly what participants have or have not agreed to; and it also makes it difficult to respect the individual preferences and autonomy of participants [5]. With the advent of legislation that protects privacy of individuals, like the General Data Protection Regulation (GDPR) in the European Union (EU), or the Protection of Personal Information (POPI) Act in South Africa, it is important to have consent from individuals specifically for sharing their health data with other researchers and/or across international borders. While asking consent for each specific use might limit re-use for new types of research in the future that we do not yet know about, including consent specifically to be re-contacted means that researchers can contact participants about new types of studies in the future. Whilst not all participants might agree to re-contact for future studies, for those that do, this provides an option to consult them directly without the researcher or an ethics review board making these important decisions on behalf of the participant but without their knowledge.

Building informed consent processes without prior experience can be daunting, so we have aimed to assist researchers by developing this template that reminds researchers of the types of consent they can request for genomics studies and assists them with suggestions for the language they might use for participant information and consent questions, whilst allowing them the freedom to include or exclude certain modules and modify the language that they use. Finally, immediate electronic capture of the consents given by participants can facilitate accurate and efficient onward sharing of data and samples according to participant preferences that can be easily electronically queried. This can replace the current common practice of unwieldy storage of paper consent forms that need to be reviewed individually to determine which data or samples can be re-used. The use of this e-consent module can thus facilitate efficient and ethical data- and sample-sharing, whilst respecting the specific preferences and choices of each participant.

Whilst this REDCap template utilises a digital approach to presenting and capturing the informed consent process, which comes with the described advantages such as improved data fidelity and streamlined databasing of participant choices, the fundamental process and material content of tiered informed consent remains consistent with current paper-based tiered informed consent processes for health genomics research [15, 25]. This point can be communicated clearly to ethics review committees assessing the use of the template for the first time. As with current practice, and as described here, it remains important to validate the informed consent process to ensure it is locally relevant, through community engagement, for example by holding community-based focus groups to evaluate local accessibility of the content. Other important inputs include training researchers in the use of the digital informed consent process to ensure high quality data collection as well as to ensure participants understand how the digital tool is being used. Ongoing data quality control can also ensure effective use and appropriate data capture with the REDCap informed consent tool. Through these approaches, participants, researchers, and ethics review boards can gain confidence that the informed consent process is operating as intended.

## Supplementary Information

**Additional file 1.** Participant information and informed consent checklist for new research study.

**Additional file 2.** **Supplementary Table 2:** List of the documents in the tiered e-consent framework Github repository.

**Additional file 3.** **Supplementary Table 1:** Additional REDCap survey customisations that were used in the tiered econsent documents.

**Additional file 4.** **Supplementary data file 2:** Example of consent dashboard.

**Additional file 5.** **Supplementary data file 3:** Consent Withdrawal Dashboard.

**Additional file 6.** **Supplementary data file 4:** Example of study population data summarised for each type of consent.

## Data Availability

The data supporting the conclusions of this article are available in the CIDRI-Africa GitHub repository, https://github.com/CIDRI-Africa/e-Consent-framework.

## Abbreviations

DNA: Deoxyribonucleic acid
e-consent: Electronic capture of consent process
IRB: Institutional review board
ID: Identification
PID: Unique record name
PDF: Portable document format
REDCap: Research Electronic Data Capture
