# Supplementary material for: An e-consent framework for tiered informed consent for human genomic research in the global south, implemented as a REDCap template
Source: BMC Med Ethics. 2022 Nov 24;23:119. doi: 10.1186/s12910-022-00860-2 (PMC9694827; doi:10.1186/s12910-022-00860-2)
Supplement: Supplementary file 1 — Additional file 1. Participant information and informed consent checklist for new research study. [file 12910_2022_860_MOESM1_ESM.pdf]

Participant information and informed consent checklist for a new research study.

1. **Steps to create the tiered consent workflow using the REDCap template:**

    1.1. Set up research study database in REDCap
    1.2. Download tiered e-consent template codebook (ConsentFramework_Data_Dictionary) and supporting documents from GitHub repository
    1.3. Import tiered e-consent template codebook in REDCap
    1.4. Use the guidance documents provided to set up and enable e-consent module in REDCap
    1.5. Submit e-consent documents to relevant institutional review board for ethics approval
    1.6. Train research staff on administering tiered e-consent
    1.7. Implement use of e-consent in new research study participant recruitment

2. **Participant information and informed consent modules to include:**

**Type of consent**	✓/ ×
Primary consent for collecting biospecimens and health data for specific disease in current study.	
Consent for access to medical records	
Consent for return of individual results	
Consent for return of individual results that are actionable and/or treatable	
Consent for return of individual results that are NOT actionable and/or treatable	
Consent for inclusion of individual data in genetic summary data	
Consent for use of genetic and health data for future studies on specific disease	
Consent for use of genetic and health data for future studies on other health conditions or related health processes	
Consent to re-contact for future studies	
Consent for use of genetic and health data in international studies	
Consent for use of genetic data in population origins and ancestry studies	
