# Supplementary material for: An e-consent framework for tiered informed consent for human genomic research in the global south, implemented as a REDCap template
Source: BMC Med Ethics. 2022 Nov 24;23:119. doi: 10.1186/s12910-022-00860-2 (PMC9694827; doi:10.1186/s12910-022-00860-2)
Supplement: Supplementary file 2 — Additional file 2. Supplementary Table 2: List of the documents in the tiered e-consent framework Github repository. [file 12910_2022_860_MOESM2_ESM.pdf]

Supplementary Table 2: List of the documents in the tiered e-consent framework Github repository.

Name in GitHub Repository (including link to document)	Document content	Use of content
ConsentFramework.xml https://github.com/CIDRI-Africa/e-Consent-framework/blob/main/ConsentFramework_2021-09-29_1108.REDCap.xml	XML file which contains the entire tiered e-consent project metadata	This can be imported into REDCap to set up a clone of the project.
ConsentFramework_Data_Dictionary https://github.com/CIDRI-Africa/e-Consent-framework/blob/main/ConsentFramework_DataDictionary_2021-09-29.csv	CSV file which contains all the tiered e-consent variables	This is the codebook that can be used to set up a new instance of the e-consent framework
ConsentFramework_All_Documents https://github.com/CIDRI-Africa/e-Consent-framework/blob/main/ConsentFramework_Allforms_20210929.pdf	PDF of example copies of the different documents generated used the tiered e-consent framework	Example output
Instrument index.xls https://github.com/CIDRI-Africa/e-Consent-framework/blob/main/Instrument%20index%2020210929.xlsx	List of all data capture instruments available in the e-consent	Shows which instruments are essential and which are optional when implementing your own instance of the framework
Set up guide.doc https://github.com/CIDRI-Africa/e-Consent-framework/blob/main/Set-up%20guide%2020200204.docx	A word document	Step by step guide on how to set up REDCap and use the tiered e-consent feature
Diabetes study example.pdf https://github.com/CIDRI-Africa/e-Consent-framework/blob/main/Diabetes_study_example.pdf	A PDF document	An example of a tiered e-consent document showing the final output from the different data capture tools.
