# Supplementary material for: An e-consent framework for tiered informed consent for human genomic research in the global south, implemented as a REDCap template
Source: BMC Med Ethics. 2022 Nov 24;23:119. doi: 10.1186/s12910-022-00860-2 (PMC9694827; doi:10.1186/s12910-022-00860-2)
Supplement: Supplementary file 3 — Additional file 3. Supplementary Table 1: Additional REDCap survey customisations that were used in the tiered econsent documents. [file 12910_2022_860_MOESM3_ESM.pdf]

Supplementary Table 1: Additional REDCap survey customisations that were used in the tiered e-consent documents

**Customisation**	**Utility of customisation**
Set a Custom Record Label	Allows another variable to the appended to the system generated record name to aid in ease of identification of individual participants records
Designate a Secondary Unique Field	A unique constraint value which cannot be duplicated and will be checked in real time to ensure that is not shared by another record e.g., participant study ID
Require a reason when making changes to existing records	Require users to enter a reason (200 characters max) in a text box when making any data changes to an already existing record on a data collection instrument. The prompt is triggered when clicking the Save button on the page. Any 'reasons' entered can then be viewed anytime afterward on the Logging page. his feature is only triggered when adding, editing, or deleting data for an instrument that contains previously collected data for one or more fields on the instrument.
Display the Today/Now button for all date and time fields on forms/surveys?	If enabled, a 'Today' button will be displayed to the right of all date fields, and a 'Now' button will be displayed to the right of all time, datetime, and datetime_seconds fields. Clicking the button will automatically set the field's value with the current date or time.
Enable the File Version History for 'File Upload' fields?	If a new version of a file needs to be uploaded for the field, instead of deleting the current file before adding the new one, you may simply upload a new file (via the 'Upload new version' link), in which all older versions will be kept and will be accessible

	for viewing/download in the Data History popup for the field. This feature provides the convenience of accessing older versions of the file instead of having to delete them. (Note: Older versions of a file will not be accessible anywhere else in the project except the Data History popup.
Enable the Data History popup for all data collection instruments?	If enabled, an icon will appear next to every field on a data collection instrument. When the icon is clicked, the history of all data entered into that field for that record will be listed chronologically and will display all previous values, who changed the value at each instance, and the time it was changed.
Enable the Field Comment Log or Data Resolution Workflow (Data Queries)?	The Field Comment Log (enabled by default) allows users to leave comments for any given field on a data entry form by clicking the balloon icon next to the field. All comments can also be viewed, searched, and downloaded on the Field Comment Log page.
