# Supplementary material for: An e-consent framework for tiered informed consent for human genomic research in the global south, implemented as a REDCap template
Source: BMC Med Ethics. 2022 Nov 24;23:119. doi: 10.1186/s12910-022-00860-2 (PMC9694827; doi:10.1186/s12910-022-00860-2)
Supplement: Supplementary file 4 — Additional file 4. Supplementary data file 2: Example of consent dashboard. [file 12910_2022_860_MOESM4_ESM.pdf]

Supplementary data file 2: Example of consent dashboard

Data Exports, Reports, and Stats

Consent dashboard for diabetes

Search

Re-enable floating table headers ?

**PID** pid	**Event Name** redcap_event_name	**Study ID Number** study_id_v2	**Date of consent** consent_date_v2	**Do you agree for us to collect these body fluid samples and your he ... might affect type 2 diabetes?** consent_data_collection_v2	**We would like to know more about your general health. Do you agree ... its to health care facilities?** consent_health_information_v2	**Do you agree for us to use your medical record number to access your health information?** consent_medical_record_number_v2	**Sometimes, what we find from our research might include new informa ... y directly affect your health?** consent_new_info_contact_v2	**Would you like us to contact you again if there is some kind of act ... elp you with the health issue?** consent_new_tx_contact_v2	**Would you like us to contact you again if there is NO kind of actio ... elp you with the health issue?** consent_no_tx_contact_v2	**Sometimes researchers combine the genetic information from everyone ... al individuals in this study)?** consent_grouped_data_v2	**Do you agree for us to use your genetic samples together with your ... t of genes on type 2 diabetes?** consent_samples_future_use_specific_pheno_v2	**Do you agree for us to use your genetic samples together with your ... related biological processes?** consent_samples_future_use_other_or_related_v2	**Sometimes what we find from a study like this might lead to new stu ... art in other research studies?** consent_future_research_contact_v2	**If yes, how would you like to be contacted?** consent_future_research_contact_type_v2	**Do you agree for us to share your DNA sample for genetic analysis t ... or other studies in the future** consent_international_research_v2	**Do you agree for us to share your DNA sample for genetic analysis i ... pulation origins and ancestry?** consent_population_origin_ancestry_v2
214	Data collection (Arm 3: Diabetes example)	T2D_001	01-09-2021	Yes (1)	Yes (1)	Yes (1)	Yes (1)	Yes (1)	Yes (1)	Yes (1)	Yes (1)	Yes (1)	Yes (1)	Email (4)	Yes (1)	Yes (1)
215	Data collection (Arm 3: Diabetes example)	T2D_002	02-09-2021	Yes (1)	Yes (1)	Yes (1)	No (0)	No (0)	No (0)	Yes (1)	Yes (1)	Yes (1)	Yes (1)	Telephone (1)	Yes (1)	Yes (1)
216	Data collection (Arm 3: Diabetes example)	T2D_003	03-09-2021	Yes (1)	No (0)		Yes (1)	Yes (1)	No (0)	Yes (1)	Yes (1)	Yes (1)	No (0)		No (0)	No (0)
217	Data collection (Arm 3: Diabetes example)	T2D_004	03-09-2021	Yes (1)	Yes (1)	Yes (1)	Yes (1)	Yes (1)	No (0)	Yes (1)	Yes (1)	Yes (1)	Yes (1)	Email (4)	Yes (1)	No (0)
218	Data collection (Arm 3: Diabetes example)	T2D_005	06-09-2021	Yes (1)	Yes (1)	Yes (1)	No (0)	No (0)	No (0)	Yes (1)	Yes (1)	Yes (1)	No (0)		No (0)	No (0)
219	Data collection (Arm 3: Diabetes example)	T2D_006	06-09-2021	Yes (1)	Yes (1)	Yes (1)	Yes (1)	Yes (1)	No (0)	Yes (1)	Yes (1)	Yes (1)	Yes (1)	Visit (3)	Yes (1)	No (0)
