# Supplementary material for: An e-consent framework for tiered informed consent for human genomic research in the global south, implemented as a REDCap template
Source: BMC Med Ethics. 2022 Nov 24;23:119. doi: 10.1186/s12910-022-00860-2 (PMC9694827; doi:10.1186/s12910-022-00860-2)
Supplement: Supplementary file 5 — Additional file 5. Supplementary data file 3: Consent Withdrawal Dashboard. [file 12910_2022_860_MOESM5_ESM.pdf]

Supplementary data file 3: Consent Withdrawal Dashboard

Data Exports, Reports, and Stats

Study withdrawal dashboard

Search

Re-enable floating table headers ?

			Please select from the list below from which part(s) of the study y ... ike to withdraw your consent										
**Study ID Number** study_id_v2	**Date** date_of_exit_v2	**Do you wish to withdraw your consent to participate in the entire study or parts of the study?** exit_type_v2	**I no longer agree for you to collect my body fluid samples and health information for this study?** partial_exit_v2___1	**I no longer agree for you to use my health information?** partial_exit_v2___2	**I no longer agree for you to use my medical record number?** partial_exit_v2___3	**I no longer agree for you to contact me again if you believe you have new information that may directly affect my health?** partial_exit_v2___4	**I no longer agree for you to contact me again if there is some kind of action or treatment that might be able to help with my health issue?** partial_exit_v2___5	**I no longer agree for you to contact me again if there is NO kind of action or treatment that might be able to help with my health issue?** partial_exit_v2___6	**I no longer agree for you to use my information when providing combined information about the whole research group?** partial_exit_v2___7	**I no longer agree for you to use my genetic samples together with my health information for other studies in the future to study the effect of genes on other health conditions or related biological processes?** partial_exit_v2___8	**I no longer agree for researchers to contact me in the future to invite me to take part in other research studies?** partial_exit_v2___9	**I no longer agree for you to share my DNA sample for genetic analysis together with my health information for international studies being done to better understand type 2 diabetes?** partial_exit_v2___10	**I no longer agree for you to share my DNA sample for genetic analysis in other research studies about population origins and ancestry?** partial_exit_v2___11
			Unchecked (0)	Unchecked (0)	Unchecked (0)	Unchecked (0)	Unchecked (0)	Unchecked (0)	Unchecked (0)	Unchecked (0)	Unchecked (0)	Unchecked (0)	Unchecked (0)
T2D_001	29-09-2021	Partial withdrawal (2)	Unchecked (0)	Unchecked (0)	Unchecked (0)	Unchecked (0)	Unchecked (0)	Unchecked (0)	Unchecked (0)	Unchecked (0)	Unchecked (0)	Checked (1)	Checked (1)
T2D_002			Unchecked (0)	Unchecked (0)	Unchecked (0)	Unchecked (0)	Unchecked (0)	Unchecked (0)	Unchecked (0)	Unchecked (0)	Unchecked (0)	Unchecked (0)	Unchecked (0)
T2D_003	29-09-2021	Complete withdrawal (1)	Unchecked (0)	Unchecked (0)	Unchecked (0)	Unchecked (0)	Unchecked (0)	Unchecked (0)	Unchecked (0)	Unchecked (0)	Unchecked (0)	Unchecked (0)	Unchecked (0)
T2D_004			Unchecked (0)	Unchecked (0)	Unchecked (0)	Unchecked (0)	Unchecked (0)	Unchecked (0)	Unchecked (0)	Unchecked (0)	Unchecked (0)	Unchecked (0)	Unchecked (0)
T2D_005			Unchecked (0)	Unchecked (0)	Unchecked (0)	Unchecked (0)	Unchecked (0)	Unchecked (0)	Unchecked (0)	Unchecked (0)	Unchecked (0)	Unchecked (0)	Unchecked (0)
T2D_006			Unchecked (0)	Unchecked (0)	Unchecked (0)	Unchecked (0)	Unchecked (0)	Unchecked (0)	Unchecked (0)	Unchecked (0)	Unchecked (0)	Unchecked (0)	Unchecked (0)
T2D_007			Unchecked (0)	Unchecked (0)	Unchecked (0)	Unchecked (0)	Unchecked (0)	Unchecked (0)	Unchecked (0)	Unchecked (0)	Unchecked (0)	Unchecked (0)	Unchecked (0)
