# Supplementary material for: An e-consent framework for tiered informed consent for human genomic research in the global south, implemented as a REDCap template
Source: BMC Med Ethics. 2022 Nov 24;23:119. doi: 10.1186/s12910-022-00860-2 (PMC9694827; doi:10.1186/s12910-022-00860-2)
Supplement: Supplementary file 6 — Additional file 6. Supplementary data file 4: Example of study population data summarised for each type of consent. [file 12910_2022_860_MOESM6_ESM.pdf]

Supplementary Data File 4: Example of study population data summarised for each type of consent

Do you agree for us to share your DNA sample for genetic analysis together with your health information for International studies being done to better understand type 2 diabetes? Your genetic data and health data may be shared with other international researchers for other studies in the future *(consent_international_research_v2)* Refresh Plot | View as Bar Chart

Total Count (N)	Missing*	Unique
21	0 (0.0%)	2

Counts/frequency: Yes (15, 71.4%), No (6, 28.6%)

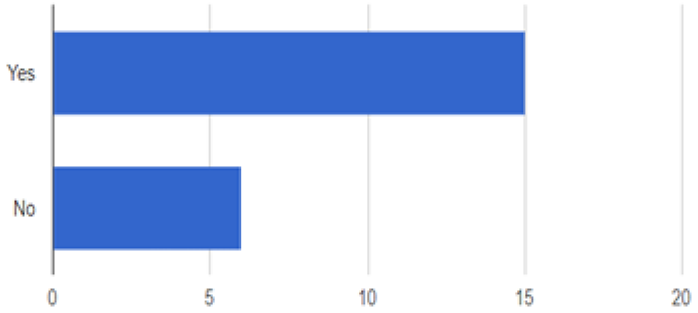

Download image
